# RAFF-4, Magnetization Transfer and Diffusion Tensor MRI of Lysophosphatidylcholine Induced Demyelination and Remyelination in Rats

**DOI:** 10.3389/fnins.2021.625167

**Published:** 2021-03-04

**Authors:** Klara Holikova, Hanne Laakso, Raimo Salo, Artem Shatillo, Antti Nurmi, Martin Bares, Jiri Vanicek, Shalom Michaeli, Silvia Mangia, Alejandra Sierra, Olli Gröhn

**Affiliations:** ^1^Department of Medical Imaging, St. Anne’s University Hospital Brno and Faculty of Medicine, Masaryk University, Brno, Czechia; ^2^A.I. Virtanen Institute for Molecular Sciences, University of Eastern Finland, Kuopio, Finland; ^3^Charles River Laboratories, Kuopio, Finland; ^4^First Department of Neurology, St. Anne’s University Hospital Brno and Faculty of Medicine, Masaryk University, Brno, Czechia; ^5^Department of Neurology, School of Medicine, University of Minnesota, Minneapolis, MN, Untied States; ^6^Center for Magnetic Resonance Research, University of Minnesota, Minneapolis, MN, Untied States

**Keywords:** myelin, demyelination, remyelination, MRI, diffusion, rotating frame relaxation

## Abstract

Remyelination is a naturally occurring response to demyelination and has a central role in the pathophysiology of multiple sclerosis and traumatic brain injury. Recently we demonstrated that a novel MRI technique entitled Relaxation Along a Fictitious Field (RAFF) in the rotating frame of rank n (RAFFn) achieved exceptional sensitivity in detecting the demyelination processes induced by lysophosphatidylcholine (LPC) in rat brain. In the present work, our aim was to test whether RAFF4, along with magnetization transfer (MT) and diffusion tensor imaging (DTI), would be capable of detecting the changes in the myelin content and microstructure caused by modifications of myelin sheets around axons or by gliosis during the remyelination phase after LPC-induced demyelination in the corpus callosum of rats. We collected MRI data with RAFF4, MT and DTI at 3 days after injection (demyelination stage) and at 38 days after injection (remyelination stage) of LPC (*n* = 12) or vehicle (*n* = 9). Cell density and myelin content were assessed by histology. All MRI metrics detected differences between LPC-injected and control groups of animals in the demyelination stage, on day 3. In the remyelination phase (day 38), RAFF4, MT parameters, fractional anisotropy, and axial diffusivity detected signs of a partial recovery consistent with the remyelination evident in histology. Radial diffusivity had undergone a further increase from day 3 to 38 and mean diffusivity revealed a complete recovery correlating with the histological assessment of cell density attributed to gliosis. The combination of RAFF4, MT and DTI has the potential to differentiate between normal, demyelinated and remyelinated axons and gliosis and thus it may be able to provide a more detailed assessment of white matter pathologies in several neurological diseases.

## Introduction

Myelin is essential for the proper functioning of the central nervous system. It not only accelerates the propagation of electrical impulses along myelinated fibers, but it also provides protection and nutrients to neurons (Saab and Nave, 2017). Disturbances in the integrity of myelin can cause a wide variety of motor, sensory and cognitive symptoms, and demyelination, e.g., damage or loss of myelin sheaths has been associated with several diseases including multiple sclerosis (Noseworthy et al., 2000), Alzheimer’s disease (Nasrabady et al., 2018), and traumatic brain injury (Armstrong et al., 2016a).

Remyelination is a natural regenerative response to demyelination. Both acquired and genetic demyelinations are followed by remyelination, and this has been found to play an important role especially in multiple sclerosis (Prineas and Connell, 1979; Hirano, 1989) and traumatic brain injury (Armstrong et al., 2016b). Oligodendrocytes create new myelin sheaths that cover the demyelinated axons; however, the newly formed myelin sheaths are typically thinner than the original myelin sheaths and/or may have a different structure and altered conduction properties (Zhao et al., 2005; Franklin and Ffrench-Constant, 2008). Remyelination is a key step in the patient’s recovery process, as electrical impulses propagate too slowly along demyelinated axons to allow normal brain function. Non-invasive quantitative imaging of changes in myelin content and microstructure can provide critical information about demyelination and remyelination processes and be useful for monitoring the progression of diseases and responses to treatment.

There are several methods available which can be used for imaging of demyelination, however, MRI is able to map myelin only indirectly (Heath et al., 2018). Direct detection of myelin is difficult as the movement restriction of lipid chains in the myelin bilayer causes a fast relaxation decay of the MR signal, although it may become more feasible by adopting zero echo time imaging approaches (Wilhelm et al., 2012; Seifert et al., 2017). Diffusion MRI, in particular diffusion tensor imaging (DTI), monitors the microscopic motion of water molecules that occur in brain tissues as a part of the diffusion process. As myelin sheaths restrict water diffusion, DTI can detect abnormalities in the structure of white matter, although it is not specific for the myelin compartment as many other cell structures contribute to the restriction of diffusion in tissue. Magnetization transfer (MT) MRI is an indirect method that was proposed many years ago for the detection of demyelination (Wolff and Balaban, 1989). This method utilizes the exchange of magnetization between the hydrogen nuclei of semisolid macromolecules and hydrogen protons in free water; as a consequence, semisolid tissue components such as myelin structures can modulate the MR image contrast. One limitation to the use of MT for monitoring myelin is that other macromolecular tissue components, as well as changes in the water content due to edema, also affect the MT contrast. Multi-exponential T2 can serve as a potential indicator of the myelin content in white matter. However, the relative size of the short-T2 component around 8–50 ms is defined as myelin associated water, and this has often been interpreted as the myelin content (Dula et al., 2010). While the water fraction of myelin has been found to correlate with the myelin content, the exact relationship between the short T2 component and the myelin content is not well understood (Tozer et al., 2005).

A novel rotating frame relaxation method operating in non-adiabatic regime, entitled Relaxation Along a Fictitious Field (RAFF) (Liimatainen et al., 2010, 2011) in the rotating frame of rank n (RAFFn) (Liimatainen et al., 2015), was recently presented and shown to have excellent sensitivity for myelin detection both in normal brain (Hakkarainen et al., 2016) and in demyelinated lesions induced by lysophosphatidylcholine (LPC) injections into the corpus callosum and in the dorsal tegmental tract (Lehto et al., 2017) of the rat brain and in dysmyelination (Satzer et al., 2015) in mouse brain. The correlation of relaxation time constants detected with RAFF4 (TRAFF4) with the myelin content obtained in a previous study (Lehto et al., 2017) was ascribed to the increased sensitivity of RAFFn to slow/ultra-slow motional regimes. These have correlation times of motion in the ms range (Liimatainen et al., 2015; Satzer et al., 2015; Hakkarainen et al., 2016), likely reflecting the exchange of myelin associated water as well as the conformational dynamics of methylene functional groups within myelin. The highest correlation between relaxation time constants and the myelin content was achieved with RAFF4 and RAFF5 techniques as compared to T1, T2 and conventional spin-lock rotating frame relaxation contrasts (Satzer et al., 2015; Hakkarainen et al., 2016) in the rat brain. In addition, RAFFn provides the distinct advantage of resulting in a substantially lower specific absorption rate (SAR) as compared to conventional continuous wave (CW) (Liimatainen et al., 2010, 2015).

While our previous work demonstrated the clear advantages of RAFFn in the detection of demyelination (Lehto et al., 2017), the process of remyelination was not assessed by multimodal MRI. In the present work, we hypothesize that by combining microstructural imaging, DTI, and methods specific to myelin content, RAFFn and/or MT, it is possible to characterize both the myelin content and the integrity of myelin sheaths during remyelination. To test this hypothesis, we used LPC-induced demyelination in the rat corpus callosum, and conducted a longitudinal study using multiparametric MRI data during both the acute demyelination and chronic remyelination phases and compared the results with histological findings.

## Materials and Methods

### Animal Model

A total of 26 adult male Sprague-Dawley rats (Charles River, Germany; 300–350 g) were used in this study. Rats were group housed with a 12 h light/12 h dark cycle and had *ad libitum* access to food and water. All animal procedures were approved by the Animal Ethics Committee of the Provincial Government of Southern Finland and conducted in accordance with the guidelines set by the European Commission Directive 2010/63/EEC.

All surgical procedures were done under inhalation anesthesia using 1.8–2.2% isoflurane in 30%/70% O_2_/N_2_O. To induce demyelinated lesions, stereotaxic injections of the LPC solution (volume of 1.5 μl; concentration: 10 mg/ml; L-α- lysophosphatidylcholine from egg yolk; L-4129 Sigma-Aldrich, St. Louis, United States) were administered into the corpus callosum of the rat brain with stereotactic coordinates of 0.4 mm caudal from bregma, 1.4 mm left from bregma, and 2.6 mm from the brain surface (*n* = 17). Control animals (*n* = 9) underwent the identical protocol but were injected with 1.5 μl of vehicle solution of 0.1 M sodium phosphate buffer solution instead of LPC.

### Pilot Study

A pilot study was first performed to clarify the time course of the demyelination/-remyelination process in the LPC model under our experimental conditions. It has been previously described that demyelination without an inflammatory reaction peaks at day 3 after LPC injection (Waxman et al., 1979; Lehto et al., 2017). However, it was our intention to determine the time course of remyelination. In the pilot experiment, 5 LPC rats were imaged at 7 T MRI (Bruker Pharmascan, Entlingen, Germany) with an actively decoupled quadrature receiver rat head coil and volume transmit coil pair every 2–3 days for 38 days using a high-resolution T2-weighted fast spin-echo (FSE) sequence with the following parameters: TR = 2.6 s, averages = 8, TE_*eff*_ = 42.7 ms, rare factor = 8, FOV = 25.6 × 25.6 mm^2^, matrix size = 256 × 256, number of slices = 24 and slice thickness = 0.3 mm) with total imaging time of 10 min 55 s. Immediately after the final scanning, the animals were perfused for histology.

### MRI Protocol to Study Demyelination and Remyelination

The remaining rats (*n* = 21) were imaged on day 3 after the LPC (*n* = 12) or vehicle (*n* = 9) injection, when there was already a significant demyelination without any inflammatory reaction or any signs of remyelination (Waxman et al., 1979), and again on day 38 after the injection when there should be a marked remyelination according to our pilot study. All MRI procedures were performed with the 7 T MRI system described above. The location of injections was localized using T2-weighted FSE acquisitions. The center of the imaging slice for RAFF4, MT, and DTI (middle slice), on both day 3 and day 38, was positioned to align with the center of the T2-weighted slice next (caudal) to the slice covering the injection site to exclude any mechanical damage induced by the injection.

For the relaxation and MT measurements, a FSE pulse sequence was used as the readout portion of the sequence. The parameters for the readout were TR = 4 s, TE_*eff*_ = 8.3 ms, n_*echo*_ = 8, FOV = 32.0 × 32.0 mm^2^, matrix size = 256 × 256, number of slices = 1 and slice thickness = 0.5 mm with a total acquisition time of 16 min for one relaxation time constant map.

The RAFFn method has been presented in detail previously (Liimatainen et al., 2015). Here, we used RAFF4; to generate RAFFn contrast, trains of RAFFn pulses assembled in *P*-packets (*PP*^–1^ *P*_π_ *P*_π_^–1^) were used as described before (Liimatainen et al., 2010). The duration of each RAFF4 pulse, defined as T_*p*_ = 4π/(√2ω_*1*_^max^), was set to 4.525 ms and the peak RF amplitude was γ*B*_1_ = 324 Hz. The RAFF4 pulse train durations were 0, 109, 217, 326, and 434 ms. Separate measurements were performed with and without an adiabatic full passage (AFP) inversion pulse (hyperbolic secant (HS1) pulse, T_*p*_ = 8 ms, γ*B*_1_ = 2,500 Hz) preceding the RAFFn pulse trains (Liimatainen et al., 2010).

RAFF4 was calculated by a non-linear least-squares fitting approach simultaneously on data obtained with initial -z′ and +z′ magnetization orientations (Liimatainen et al., 2010). Equation 1 was used to model the observed exponential decay and the approach to steady state,

(1) S±Z(t)=Se-Rt0,±Z-S(1-e)-RtSS

Here, S_0_ is the initial signal intensity (t = 0), R is the relaxation rate constant describing the decay, and S_*SS*_ is the steady-state intensity at t → ∞.

In acquiring MT metrics, we used the modified inversion MT protocol with two consecutive acquisitions as described previously (Mangia et al., 2011). Separate measurements were performed with the magnetization initially aligned along the +*z* axis during off-resonance irradiation, or -*z* axis to allow the signal to recover, i.e., without or with initial global inversion achieved by an adiabatic full passage (AFP) pulse, in analogy to the acquisitions with RAFF4. A square saturation pulse with γ*B*_1_ = 200 Hz was placed at 8 kHz off-resonance with an incremental duration (0.0, 0.3, 0.6, 0.9, 1.2 s). T_1s__*at*_, M_*SS*_ (steady state magnetization) and M_0_ (fully relaxed magnetization in the absence of RF), were calculated using pixel-by-pixel analysis, as described by Mangia et al. (2011). MTR was also calculated as MTR = 1–M_*SS*_/M_0_.

For DTI, segmented spin-echo EPI was used with TR = 1 s, TE = 31.8 ms, n_*shots*_ = 2, number of averages = 48, FOV = 21.3 × 14.4 mm^2^, matrix size = 170 × 115, number of slices = 5, slice thickness = 0.5 mm, b = 2,000 s/mm^2^, diffusion directions = 42 leading to a total acquisition time of 1 h 18 min. Mean diffusivity (MD), fractional anisotropy (FA), and radial and axial diffusivity (RD, AD) maps were calculated from DTI data. DTI data were corrected for motion and eddy current-induced image distortions using Explore DTI (Leemans et al., 2009). Relaxation time constants and parametric maps from MT and DTI were reconstructed from signal intensities using pixel-by-pixel fitting in MATLAB (MathWorks, Natick, MA) and FMRIB’s Software Library (FSL).

### Region-of-Interest (ROI) Analysis

All the images from both time points were co-registered to the RAFF4 images from day 3 using Advanced Normalization Tools (ANTs)^1^. Two ROIs in the corpus callosum, one contralateral and one ipsilateral to the injection site, were manually drawn on T2-weighted images in every animal and transferred to the co-registered stack of parametric maps using the Aedes software package^2^ When drawing the ROIs, we chose one slice caudally to the injection site based on the day 3 images and we used the same location on day 38. Mean values from each ROIs from every map were used in the statistical analysis. In the vehicle injected animals, the ROIs were drawn at the vehicle injection site similarly as conducted for the LPC- injected animals.

### Histological Procedures and Analysis

After the last MRI session, all animals were transcranially perfused first with 0.9% NaCl (30 ml/min, 2 min, 4°C) followed by 4% paraformaldehyde solution in 0.1 M phosphate buffer (pH 7.4) (30 ml/min, 25 min, 4°C). After perfusion, the brains were removed from the skull, and post-fixed for 4 h in 4% paraformaldehyde solution. Then, the brains were cryoprotected in 20% glycerol in 0.02 M potassium phosphate-buffered saline (pH 7.4) for 36 h, and frozen in dry ice, and stored at –70°C until sectioning.

The brains were sectioned into five series of 30 μm thick coronal sections using a sliding microtome. The first series was stored in 10% formalin at room temperature, and second to fifth series were stored in a cryoprotectant tissue-collecting solution (30% ethyleneglycol, 25% glycerol in 0.05 M PBS) at –20°C until staining.

Selected sections from the first series of sections were stained with Nissl (thionin) to assess changes in the cytoarchitecture of the corpus callosum. We stained up to 10 sections covering and exceeding the lesioned area as revealed in MRI on day 3. Consecutive sections from the second series were stained with gold chloride to assess the myeloarchitecture of the corpus callosum (Laitinen et al., 2010).

The optical density of Nissl- and myelin-stained sections was quantified in locations corresponding to the ROIs in the MRI analysis. Three consecutive sections were selected based on the MRI images where the ROI was drawn for analysis. The histological sections were selected based on anatomical landmarks, and the ROIs for optical density were drawn in the same anatomical location as in the MRI images in the ipsi- and contralateral corpus callosum. The three consecutive sections represent 450 μm in the rostral-caudal direction, which provides good coverage of the slice thickness of 500 μm in MRI. High-resolution photomicrographs of both Nissl- and myelin-stained sections of the corpus callosum were obtained using a light microscope (Zeiss Axio Imager2, White Plains, NY, United States) equipped with a digital camera (Zeiss Axiocam color 506). The whole corpus callosum area was imaged in each section by using the tile mode with an objective of 20×. Acquisition, alignment and format conversion were performed with Zen software (Blue edition, v2.6, Carl Zeiss Microscopy GmbH, United States).

The optical density (OD) on Nissl- and myelin-stained sections was quantified using ImageJ software (version 1.47, http://rsb.info.nih.gov/ij/, NIH, United States). First, the color photomicrographs were converted into 16-bit gray scale images, and then the gray scale was inverted to facilitate the interpretation of intensity values in the image to the intensities observed in the myelin-stained sections. We obtained the intensity values from each ROI from Nissl and myelin-stained sections. In order to correct for possible staining differences between sections and brains, the intensity values were corrected against the background intensity with no cell/myelin as in the cortical areas. OD was estimated as (I_ref_ – I_*cc*_)/I_ref_, and for each ROI, the OD value was the average of the three consecutive sections. The estimation of the area of demyelination was conducted on the myelin-stained sections by selecting the area with a low content of myelin ipsi- and/or contralaterally. This selection was limited to the area of demyelination included in the previously drawn ROI for intensity.

### Statistical Analysis

Data were analyzed using GraphPad Prism software (version 5.03 for Windows, La Jolla, CA, United States). Numerical results are represented as mean and standard deviation. Differences between vehicle- and LPC-injected rats were assessed using the two-sample *t*-test, and differences between ipsi- and contralateral corpus callosum within the same brain using the paired *t*-test. The contribution of myelinated axons and cell density to the MRI metrics was assessed using Pearson’s linear correlation of the ROI analysis results from MRI and OD of myelin- and Nissl-stained sections. The change of the MRI parameters between days 3 and 38 was assessed using paired-samples *t*-test separately for ipsi- and contralateral ROIs of vehicle- and LPC-injected rats. The Benjamini-Hochberg false discovery rate method was used for multiple comparison corrections, and FDR-threshold *q* < 0.05 was chosen for statistical significance (Benjamini and Hochberg, 1995).

## Results

**FIGURE 1:**
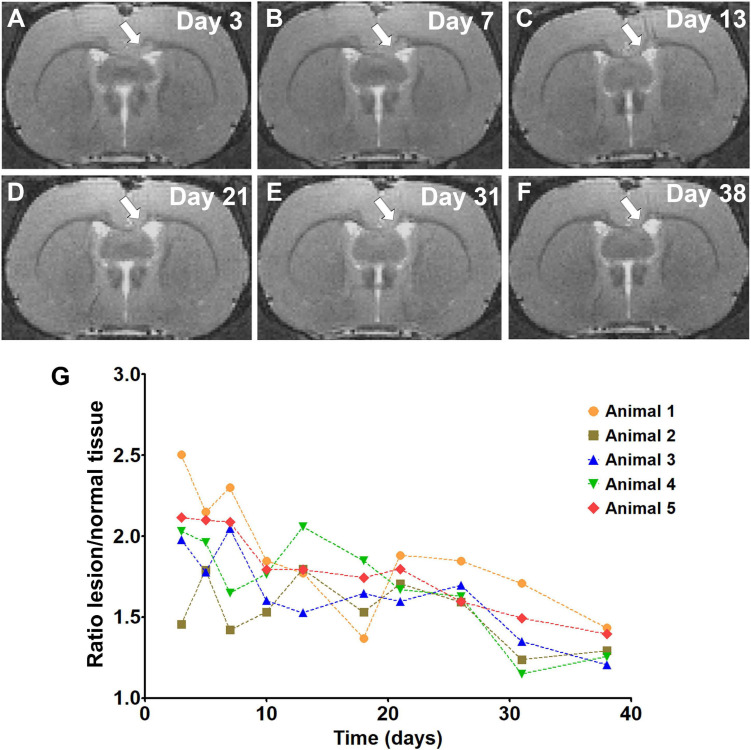
T2 weighted images showing the lesion in the corpus callosum (white arrow) and its development from day 3 to day 38 **(A–F)**. The graph represents the signal intensity ratio between lesioned and normal tissue on individual days **(G)**.

The time course of the relative signal changes in T2-weighted images after LPC injection is shown in Figure 1. This pilot experiment showed that a clear lesion could be detected on day 3 in the corpus callosum, followed by a gradual recovery of the T2-weighted signal intensity in the subsequent time points (Figure 1G). This is consistent with the demyelination/remyelination process described for the LPC model in white matter (Woodruff and Franklin, 1999). Based on this experiment, we chose day 3 as the time point for demyelination and day 38 for remyelination.

**FIGURE 2:**
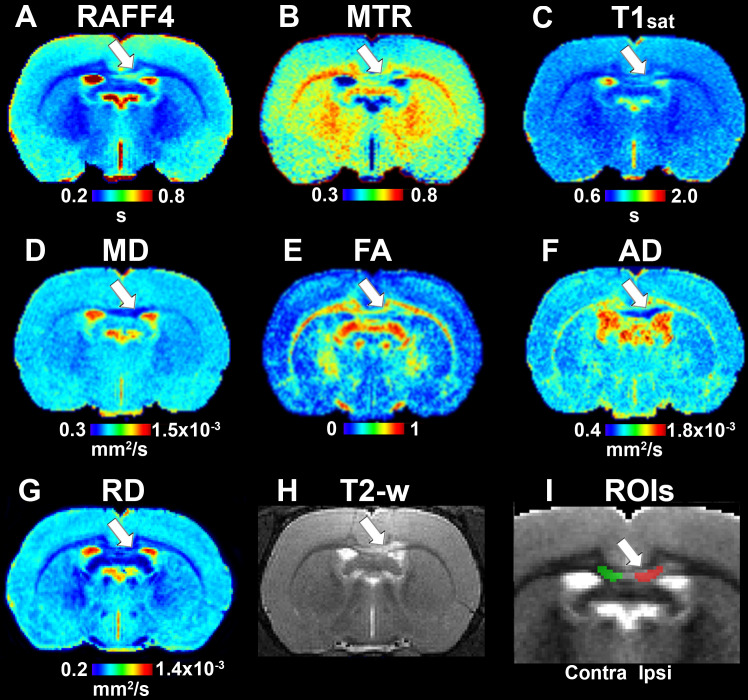
Quantitative MRI maps in the demyelination phase, on day 3: RAFF4 **(A)**, magnetization transfer ratio, MTR **(B)**, T1sat **(C)**, mean diffusivity, MD **(D)**, fractional anisotropy, FA **(E)**, axial diffusivity, AD **(F)**, radial diffusivity, RD **(G)**, T2w image with lesion **(H)** and representative example of ROIs for analy**z**ing the lesion on a grayscale RAFF4 map **(I)**. White arrow points to the lesion in the corpus callosum.

**FIGURE 3:**
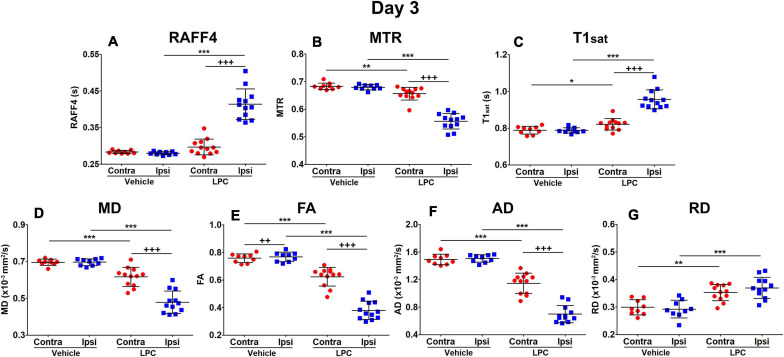
Region of interest analysis of MRI parameters in the demyelination phase, on day 3: RAFF4 **(A)**, magnetization transfer ratio, MTR **(B)**, T1sat **(C)**, mean diffusivity MD **(D)**, fractional anisotropy, FA **(E)** and axial and radial diffusivity, AD **(F),** and RD **(G)**. Values obtained from the ipsilateral and contralateral sides of LPC injected (*n* = 12) rats and from the corresponding ROI in the vehicle injected (*n* = 9) rats. Mean ± SD, paired (+) or unpaired (*) *t*-test, FDR corrected *p*-values: *<0.05, ** or ++<0.01, *** or +++<0.001.

On day 3, all the LPC animals exhibited a lesion in the MRI maps with the lesion mainly in the ipsilateral corpus callosum, but also extending to the contralateral side (Figure 2). The group-wise results and comparisons in absolute units are shown in Figure 3, while Table 1 shows relative differences and *q*-values (FDR corrected *p*-values) facilitating a comparison between modalities. The relative differences were calculated as (LPC-Vehicle)/Vehicle)^∗^100%. All MRI metrics revealed the significant and robust effect of demyelination following LPC-injected animals in the ipsilateral site (Figure 3). The largest relative differences were detected by RAFF4, FA and AD (48, –50, –54%, respectively), while MTR, T1sat and RD showed more modest (–18, 21, 26%) but still very clear changes between the demyelinated ipsilateral area and a similar area in vehicle treated animals (Table 1). The contralateral side also showed statistically significant but smaller changes between LPC and vehicle injected animals. Diffusion parameters, especially AD, FA and RD (–16, –22, 18%) were most sensitive at detecting the contralateral changes; these were caused most likely by the diffusion of LPC from the ipsilateral side to the contralateral side.

**TABLE 1:** Relative differences with statistical significances in the MRI metrics.

			RAFF4	MTR	T1sat	MD	FA	AD	RD
Day 3	Ipsi	%	48.01	–18.16	21.33	–31.18	–50.51	–53.55	26.24
		t	9.53	–12.57	9.44	–10.29	–15.89	–18.11	4.86
		q	6.12e–08	1.10e–09	6.12e–08	2.33e–08	2.77e–11	5.35e–12	0.00017
	Contra	%	4.86	4.13	–3.92	–11.43	–17.80	–23.14	17.84
		t	1.88	–3.21	2.71	–4.41	–5.51	–6.39	4.25
		q	0.088	0.0061	0.018	0.00044	6.86e–05	1.58e–05	0.00061
Day 38	Ipsi	%	17.02	–7.06	9.99	1.27	–22.33	–15.91	44.67
		t	6.03	–4.88	5.49	1.03	–5.39	–5.11	5.81
		q	2.94e–05	0.00017	6.86e–05	0.33	7.89e–05	0.00012	4.23e–05
	Contra	%	0.68	–1.11	2.78	–3.02	–17.54	–16.39	30.48
		t	0.40	–1.282	2.52	–1.85	–5.18	–5.30	4.98
		q	0.70	0.23	0.025	0.089	0.00011	8.71e–05	0.00015
Day 38 - Day 3	LPC Ipsi	%	–22.90	15.81	–12.07	42.14	60.16	75.64	12.02
		t	–7.18	10.22	–7.11	12.33	6.96	12.70	1.85
		q	9.16e–05	5.57e–06	9.16e–05	1.24e–06	9.54e–05	1.24e–06	0.12
	LPC Contra	%	–7.86	5.00	–4.72	6.78	1.33	7.19	7.41
		t	–4.65	4.67	–3.69	2.07	0.077	1.19	2.05
		q	0.0016	0.0016	0.0077	0.095	0.97	0.33	0.095
	Vehicle Ipsi	%	–3.30	1.85	–3.21	–4.53	–0.32	–4.94	–2.31
		t	–6.58	2.81	–5.79	–3.48	–0.23	–2.06	–0.68
		q	0.00060	0.043	0.0013	0.017	0.89	0.10	0.58
	Vehicle Contra	%	–4.27	1.92	–3.58	–3.12	0.13	–2.81	–2.83
		t	–9.84	2.72	–5.63	–2.36	–0.0051	–1.02	–0.74
		q	6.69e–05	0.046	0.0014	0.075	1.00	0.41	0.56

*Percentage on day 3 and day 38: ((LPC-Vehicle)/Vehicle)*100%. q value is the p-value that has been adjusted for the false discovery rate. Percentage on day 38 vs. day 3: ((day 38 - day 3)/day 3)*100 LPC and vehicle ipsi- and contralaterally. q-value on day 38 vs. day 3: (value on day 38-value on day 3) vs. zero.

**FIGURE 4:**
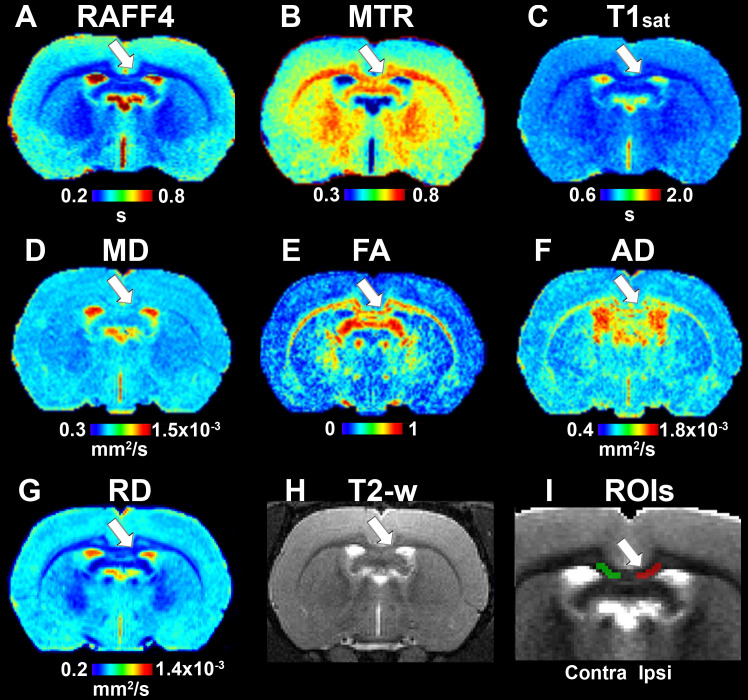
Quantitative MRI maps in the remyelination phase, on day 38. Relaxation time constant map of RAFF4 **(A)**, magnetization transfer ratio, MTR **(B)**, T1sat **(C)**, mean diffusivity, MD **(D)**, fractional anisotropy, FA **(E)**, axial diffusivity, AD **(F)**, radial diffusivity, RD **(G)**, T2w image with the lesion **(H)** and a representative example of ROIs for analyzing lesion on a grayscale RAFF4 map **(I)**. White arrow points to the lesion in the corpus callosum.

**FIGURE 5:**
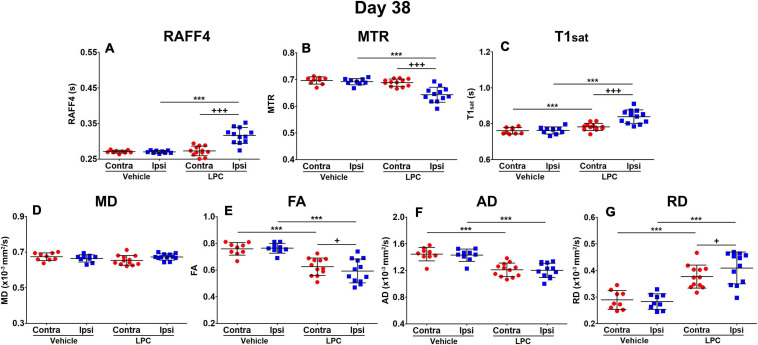
Region of interest analysis of MRI parameters in the remyelinization phase, on day 38: RAFF4 **(A)**, magnetization transfer ratio, MTR **(B)**, T1sat **(C)**, mean diffusivity MD **(D)**, fractional anisotropy, FA **(E)** and axial and radial diffusivity, AD **(F)** and RD **(G)**. Values obtained from the ipsilateral and contralateral side of LPC injected (*n* = 12) rats and from the corresponding ROI in the vehicle injected (*n* = 9) rats. Mean ± *SD*, paired (+) or unpaired (*) *t*-test, FDR corrected *p*-values: + < 0.05, *** or +++<0.001.

On day 38, all the LPC-injected animals revealed at least a partial recovery of the lesion in the MRI maps (Figure 4). Nonetheless, significant differences were still observed on day 38 between LPC and vehicle injected animals in the ipsilateral side in all other MRI metrics except the MD (Figure 5). When comparing MRI outcomes on day 3 (demyelination) to day 38 (remyelination), significant differences were detected in all MRI metrics (Table 1). In particular, the recovery toward normal values on the ipsilateral side of the LPC injected animals was detected with RAFF4 (from 48 to 17%, difference in ipsilateral side of LPC rats, from day 3 to day 38), MTR (from –18 to –7%), T1sat (from 21 to 10%), MD (from –31 to 1%), FA (from –51 to –22%), AD (from –54 to –16%). Furthermore, RD displayed a further robust increase (from 26 to 45%) from days 3 to 38.

**FIGURE 6:**
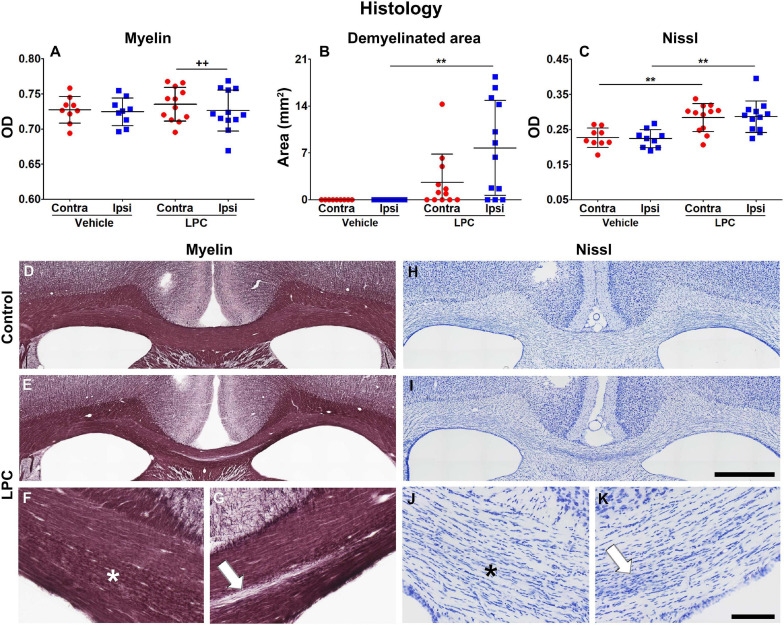
Histologic assessment of the myelin and Nissl stainings at 38 days after vehicle or LPC injection. OD **(A)** and demyelinated area **(B)** analyses of the myelin-stained sections, and OD analysis of the Nissl-stained **(C)** sections. Values were obtained from the ipsi- and contralateral corpus callosum of vehicle- (*n* = 9) and LPC-injected (*n* = 12) rats. Results are shown as mean ± *SD*. The unpaired *t*-test compared the same hemispheres between vehicle- and LPC-injected rats (**p < 0.01), and the paired *t*-test ipsi- and contralateral hemispheres within the same animals (^++^*p* < 0.01). Photomicrographs of vehicle- and LPC-injected animals in myelin **(D–G)** and Nissl **(H–K)** stains of representative rats. The white arrow points to the ongoing demyelinated area and the presence of gliosis, and the asterisk indicates the area with ongoing myelin alterations accompanied by gliosis. Scale bar: 1 mm **(D,E,H,I)** and 200 μm **(F,G,J,K)**.

Figure 6 shows the quantitative assessment of the histological results as well as representative examples of myelin- and Nissl-stained sections from vehicle- and LPC-injected animals. On day 38, the optical density (OD) analysis on myelin-stained sections revealed a small but significant decrease in the myelin content when comparing the ipsi- and contralateral ROIs in the corpus callosum in the LPC-injected brains (*q* = 0.02) (Figure 6A). We found a significant increase of the demyelinated area in animals after LPC injection in comparison to vehicle animals, ipsilaterally (*q* = 0.0085) but not contralaterally (*q* = 0.11) (Figure 6B). The demyelinated area was small as compared to the total area of the ROI analyzed in the OD analysis; these results demonstrate that the remyelination was well advanced but not completed at 38 days after the injection (Figures 6D–G). Additionally, we found that myelin alterations were taking place along the corpus callosum structure, which may be an indication of ongoing remyelinating processes (Figure 6F).

The analysis on Nissl-stained sections revealed increased cell density, which can be attributed to gliosis. The OD analysis on Nissl-stained sections showed that values in both ipsi- (*q* = 0.0032) and contralateral (*q* = 0.0085) ROIs of the corpus callosum significantly increased when comparing vehicle and LPC animals (Figure 6C). The increased cell density area overlapped with the demyelinated area (Figures 6G,K) and myelin alterations (Figures 6F,J) observed in myelin staining. These results demonstrate that the persistent demyelination was accompanied by inflammatory processes which were still ongoing at 38 days after the LPC infection.

None of the MRI parameters correlated with the OD of myelin staining in the lesion area in the remyelination phase, however, RD, FA and AD correlated with the OD assessed with Nissl staining (*q* < 0.05) (Table 2).

**TABLE 2:** Correlation between MRI metrics and the OD of myelin- and Nissl-stained sections.

	Myelin OD		Nissl OD	

	R	*p*	R	*p*
AD	–0.18086	0.25171	–0.63062	7.5945e–06
FA	–0.14081	0.37376	–0.67323	1.0335e–06
RD	0.13148	0.40656	0.64884	3.3607e–06
MD	–0.13111	0.40787	–0.071477	0.65284
MTR	0.014335	0.92821	–0.29864	0.054724
RAFF4	0.036727	0.81738	0.2594	0.097141
T1SAT	0.086483	0.58604	0.36175	0.018572

*Critical p-value (q < 0.05): 7.5945e–06.*

## Discussion

In the present work, we investigated the capabilities of quantitative RAFF4, MTR, T1sat and DTI metrics to detect LPC-induced demyelination and remyelination in rat brain corpus callosum. We confirmed the previously demonstrated high sensitivity of RAFF4, MTR, and DTI for detecting demyelination (Lehto et al., 2017). This is the first time when RAFF4 was tested for investigating the myelination status during the remyelination phase. Our main finding was that the remyelination phase was associated with a partial recovery of RAFF4, MTR, and T1sat, FA and AD, while RD remained abnormally high and MD showed a complete recovery on day 38 after LPC injection, i.e., a time point when there was histological evidence of marked remyelination and gliosis.

Our results confirmed the sensitivity of RAFF4 and MTR to detect demyelination at 3 days after the LPC injection into the corpus callosum when only mild gliosis was present (Lehto et al., 2017). The demyelination phase was also associated with a distinct pattern in the DTI metric’s changes, namely decreases in FA, AD, and MD, and an increase in RD. In our previous study, the LPC induced demyelination in the corpus callosum was characterized by a clearly decreased myelin content as detected by myelin staining. However, in that study we also observed some remaining disorganized pockets in the myelin sheaths with myelin debris being evident in electron microscopy (Lehto et al., 2017). In the present experiments, the pattern of changes in DTI metrics in demyelination phase, was mostly consistent with our previous work, however, now we did find increased RD, a parameter which was unchanged in our previous study. The present finding is in agreement with the general view that increased RD is an indication of demyelination (Song et al., 2005). The difference to the previous study may originate from differences in LPC patches leading to more severe demyelination. This is also consistent with the somewhat relatively larger changes in RAFF4 and MTR observed in the present study as compared to those reported by Lehto et al. (2017).

The remyelination phase was characterized by a close-to-normal myelin content as confirmed by OD analysis of myelin-stained histological sections. Unlike on day 3, when only very mild gliosis was present, on day 38 increased cellular density was detected in Nissl staining, likely due to gliosis. As increased cellularity affects relaxation, MT and diffusion, this makes the interpretation of the results more complicated, resembling more realistically the human pathology where myelin damage typically triggers gliosis, and thus these pathological features overlap. At the late time point, we observed a recovery of RAFF4 toward the normal values measured in the healthy tissue, which is consistent with remyelination. It has been shown that RAFF4 is sensitive to the correlation time regime in the ms-range (Satzer et al., 2015; Hakkarainen et al., 2016), which likely corresponds to exchange and dipolar interactions of myelin and water as well as dipolar interaction with methylene groups. Therefore, the high sensitivity of RAFF4 to myelin, also during the remyelination phase, was expected.

MT showed a similar recovery toward baseline as RAFF4. However, the relative difference to controls was smaller than in RAFF4, reflecting its lower sensitivity to myelination changes in the demyelination phase. Previously, RAFF4 had been shown to correlate with myelin density to a greater extent than MT in normal brain (Hakkarainen et al., 2016) and in LPC-induced demyelinated lesions in dorsal tegmental tract (dtg) of the rat brain (Lehto et al., 2017). It should be emphasized, however, that there is a distinct difference between relaxation mechanisms during RAFF4 and MT. RAFF4 is a rotating frame method operating in the rotating frame of rank 4, and thus has contributions from longitudinal, T1r, and transverse, T2r, relaxation pathways (Liimatainen et al., 2015). In addition to anisochronous and isochronous exchange and dipolar interactions contributing to RAFF4, RAFF4 share cross-relaxation pathways with MT (van Zijl et al., 2018). Therefore, these two techniques provide only partially overlapping information when characterizing tissue integrity. This substantial distinction in the relaxation mechanisms contributing to RAFF4 and MT is reflected in the differential sensitivity of RAFF4 and MT to demyelination, dismyelination and remyelination processes in the brain (Satzer et al., 2015). It is also worth noting that RAFFn offers the possibility of achieving the desired fictitious field by making use of a frequency swept pulse which improves the flexibility in handling SAR issues in human applications (Liimatainen et al., 2015).

The pattern of changes detected in DTI metrics in the remyelination phase was likely conveying information from multiple factors including the thickness and microstructure of the myelin sheaths as well as the cell density. The partial recoveries of FA and AD are similar to those detected with RAFF4 and may reflect the rebuilding of myelin sheaths and the clearance of the myelin debris. The increase in RD is consistent with the fact that the remyelinated sheaths are structurally different from intact myelin sheaths (Raine, 1984; Oluich et al., 2012; Podbielska et al., 2013; Pfeiffer et al., 2019), i.e., they are likely more permeable to water. MD was the only MRI parameter that returned to the normal level on day 38. It is well known from cancer studies that MD inversely correlates with the cellularity of the tissue (Chenevert et al., 2000) and therefore the increased cellularity due to gliosis likely contributes to the pseudo-normalization of MD. The extension to more complex diffusion MRI models has the potential to extract more specific information related to these processes (Luo et al., 2019).

MRI changes were also detected on the contralateral side of the injection between LPC and vehicle injected animals. This is likely attributable to diffusion of LPC along axons in corpus callosum such that LPC reached also the contralateral side. Interestingly, changes in cell density in Nissl, attributed to gliosis, were pronounced on the contralateral side on day 38, probably explaining the higher sensitivity of diffusion changes than were evident with RAFF4 or MT. None of the MRI parameters correlated significantly with optical density of myelin staining in the remyelination phase. This is likely because the optical densities were close to normal in the lesioned area and therefore there was a narrow range of values both for MRI and optical density. This, together with confounding effect of gliosis on MRI parameters, explains the non-significant correlation values between MRI parameters and myelin density in the remyelination phase, even though there was an evident recovery of MRI parameters, especially RAFF4 and MTR, from demyelination values. The influence of gliosis on diffusion metrics is consistent with the earlier reports of Budde et al. (2011). Consistently, we observed a correlation between cellularity in Nissl staining and diffusion parameters but not with RAFF4 or MT parameters, further emphasizing the different sensitivities of these techniques to detect myelination and cellularity.

One limitation of our study is that in spite of careful manual alignment of histology with MRI by an expert in the field, the partial volume effect and the challenge of selecting the same ROIs in MRI and histology could have influenced our results. In addition, the limited sampling in histology vs. the slice thickness in MRI may have affected our assessments of the correlations.

## Conclusion

Our data confirms the sensitivity of RAFF4 and MT for detecting the myelin content in demyelinated lesions, but now reveals that remyelination is associated with a recovery of RAFF4 and MT toward normal values. DTI metrics displayed a distinct pattern of changes in the remyelination phase, likely reflecting on-going changes not only in the myelin content but also in the architecture of the myelin sheaths as well as the presence of gliosis. The combination of RAFF4, MT and DTI has the potential to differentiate between normal, demyelinated and remyelinated axonal bundles and gliosis, thus making possible a unique non-invasive characterization of white matter pathologies in several neurological diseases. Further studies will be required to evaluate the sensitivity of multiple MRI modalities to detect remyelination in areas with more isotropic fiber distributions, where RAFF4 has demonstrated its superiority over DTI (Lehto et al., 2017).

## Data Availability Statement

The raw data supporting the conclusions of this article will be made available by the authors, without undue reservation.

## Ethics Statement

The animal study was reviewed and approved by the Animal Ethics Committee of the Provincial Government of Southern Finland.

## Author Contributions

KH, HL, and RS participated in the design of the work, acquisition, analysis, interpretation of data, and preparing the manuscript. AS, AN, MB, and JV participated in the design of the work and preparing the manuscript. ShM and SiM participated in the design of the work, interpretation of the data and preparing the manuscript. AS and OG participated in the design of the work, analysis, interpretation of data and preparing the manuscript. All authors contributed to the article and approved the submitted version.

## Conflict of Interest

The authors declare that the research was conducted in the absence of any commercial or financial relationships that could be construed as a potential conflict of interest.

## Footnotes

1 http://stnava.github.io/ANTs/

2 http://aedes.uef.fi

## Abbreviations

MRI: magnetic resonance imaging
MR: magnetic resonance
DTI: diffusion tensor imaging
MT: magnetization transfer
RAFF: relaxation along a fictitious field
MTR: magnetization transfer ratio
LPC: lysophosphatidylcholine
MD: mean diffusivity
FA: fractional anisotropy
RD: radial diffusivity
AD: axial diffusivity
FSL: FMRIB’s Software Library
ROI: region of interest
OD: optical density.
