# Corrigendum: RAFF-4, Magnetization Transfer and Diffusion Tensor MRI of Lysophosphatidylcholine Induced Demyelination and Remyelination in Rats

**DOI:** 10.3389/fnins.2021.723831

**Published:** 2021-07-09

**Authors:** Klara Holikova, Hanne Laakso, Raimo Salo, Artem Shatillo, Antti Nurmi, Martin Bares, Jiri Vanicek, Shalom Michaeli, Silvia Mangia, Alejandra Sierra, Olli Gröhn

**Affiliations:** ^1^Department of Medical Imaging, St. Anne's University Hospital Brno and Faculty of Medicine, Masaryk University, Brno, Czechia; ^2^A.I. Virtanen Institute for Molecular Sciences, University of Eastern Finland, Kuopio, Finland; ^3^Charles River Laboratories, Kuopio, Finland; ^4^First Department of Neurology, St. Anne's University Hospital Brno and Faculty of Medicine, Masaryk University, Brno, Czechia; ^5^Department of Neurology, School of Medicine, University of Minnesota, Minneapolis, MN, United States; ^6^Center for Magnetic Resonance Research, University of Minnesota, Minneapolis, MN, United States

**Keywords:** myelin, demyelination, remyelination, MRI, diffusion, rotating frame relaxation

In the original article, there was a mistake in Figures 2 and 4 as published. In both figures, radial diffusivity (RD) maps were accidently changed to incorrect ones in the final phase of Figure production. The corrected Figures 2 and 4 below. All quantitative analysis and data interpretation was performed using correct RD maps.

**Figure 2:**
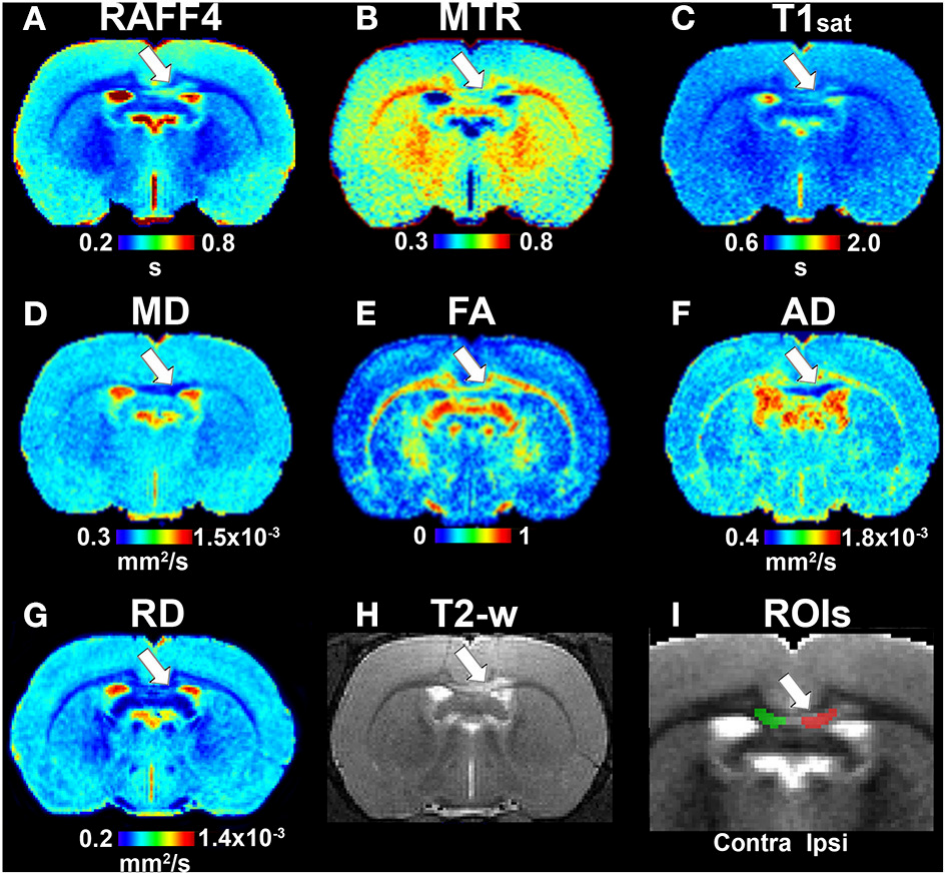
Quantitative MRI maps in the demyelination phase, on day 3: RAFF4 **(A)**, magnetization transfer ratio, MTR **(B)**, T1sat **(C)**, mean diffusivity, MD **(D)**, fractional anisotropy, FA **(E)**, axial diffusivity, AD **(F)**, radial diffusivity, RD **(G)**, T2w image with lesion **(H)** and representative example of ROIs for analyzing the lesion on a grayscale RAFF4 map **(I)**. White arrow points to the lesion in the corpus callosum.

**Figure 4:**
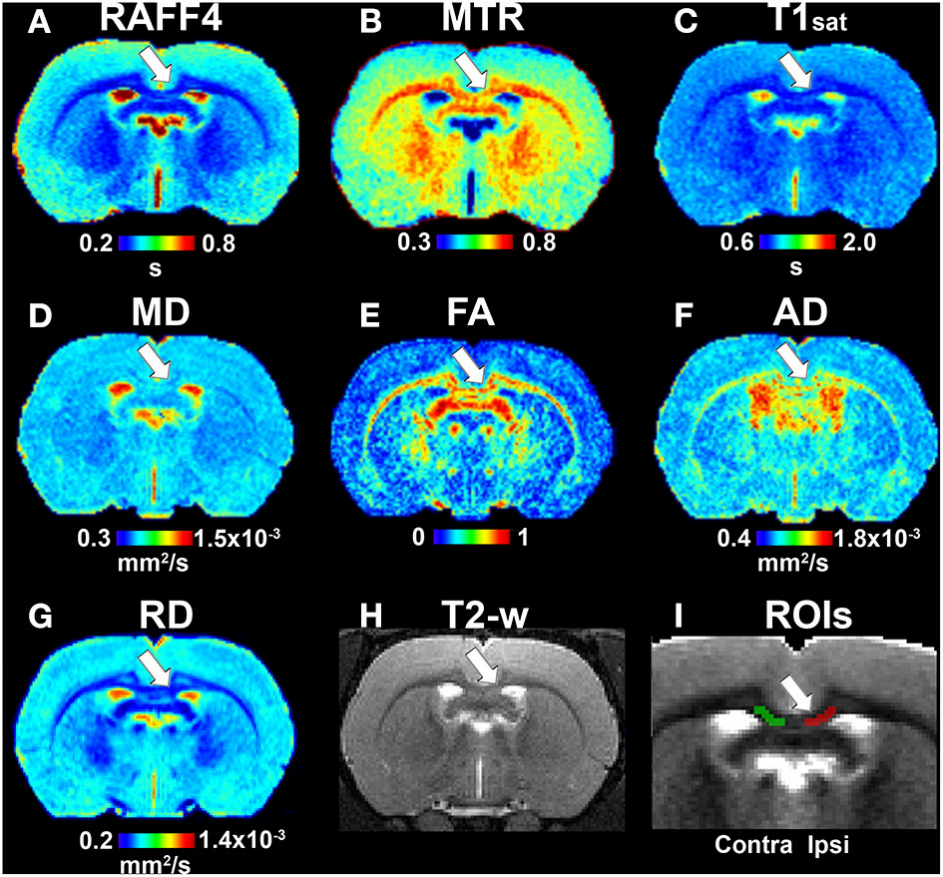
Quantitative MRI maps in the remyelination phase, on day 38. Relaxation time constant map of RAFF4 **(A)**, magnetization transfer ratio, MTR **(B)**, T1sat **(C)**, mean diffusivity, MD **(D)**, fractional anisotropy, FA **(E)**, axial diffusivity, AD **(F)**, radial diffusivity, RD **(G)**, T2w image with the lesion **(H)** and a representative example of ROIs for analyzing lesion on a grayscale RAFF4 map **(I)**. White arrow points to the lesion in the corpus callosum.

The authors apologize for this error and state that this does not change the scientific conclusions of the article in any way. The original article has been updated.

